# Designing feedback processes in the workplace-based learning of undergraduate health professions education: a scoping review

**DOI:** 10.1186/s12909-024-05439-6

**Published:** 2024-04-23

**Authors:** Javiera Fuentes-Cimma, Dominique Sluijsmans, Arnoldo Riquelme, Ignacio Villagran, Lorena Isbej, María Teresa Olivares-Labbe, Sylvia Heeneman

**Affiliations:** 1 Department of Health Sciences, Faculty of Medicine, Pontificia Universidad Católica de Chile, Avenida Vicuña Mackenna 4860, Macul, Santiago, Chile; 2 School of Health Professions Education, Maastricht University, Maastricht, Netherlands; 3 Rotterdam University of Applied Sciences, Rotterdam, Netherlands; 4 Centre for Medical and Health Profession Education, Department of Gastroenterology, Faculty of Medicine, Pontificia Universidad Católica de Chile, Santiago, Chile; 5 School of Dentistry, Faculty of Medicine, Pontificia Universidad Católica de Chile, Santiago, Chile; 6 Sistema de Bibliotecas UC (SIBUC), Pontificia Universidad Católica de Chile, Santiago, Chile; 7 Department of Pathology, Faculty of Health, Medicine and Health Sciences, Maastricht University, Maastricht, Netherlands

**Keywords:** Clinical clerkship, Feedback, Feedback processes, Feedforward, Formative feedback, Health professions, Undergraduate medical education, Undergraduate healthcare education, Workplace learning

## Abstract

**Background:**

Feedback processes are crucial for learning, guiding improvement, and enhancing performance. In workplace-based learning settings, diverse teaching and assessment activities are advocated to be designed and implemented, generating feedback that students use, with proper guidance, to close the gap between current and desired performance levels. Since productive feedback processes rely on observed information regarding a student's performance, it is imperative to establish structured feedback activities within undergraduate workplace-based learning settings. However, these settings are characterized by their unpredictable nature, which can either promote learning or present challenges in offering structured learning opportunities for students. This scoping review maps literature on how feedback processes are organised in undergraduate clinical workplace-based learning settings, providing insight into the design and use of feedback.

**Methods:**

A scoping review was conducted. Studies were identified from seven databases and ten relevant journals in medical education. The screening process was performed independently in duplicate with the support of the StArt program. Data were organized in a data chart and analyzed using thematic analysis. The feedback loop with a sociocultural perspective was used as a theoretical framework.

**Results:**

The search yielded 4,877 papers, and 61 were included in the review. Two themes were identified in the qualitative analysis: (1) The organization of the feedback processes in workplace-based learning settings, and (2) Sociocultural factors influencing the organization of feedback processes. The literature describes multiple teaching and assessment activities that generate feedback information. Most papers described experiences and perceptions of diverse teaching and assessment feedback activities. Few studies described how feedback processes improve performance. Sociocultural factors such as establishing a feedback culture, enabling stable and trustworthy relationships, and enhancing student feedback agency are crucial for productive feedback processes.

**Conclusions:**

This review identified concrete ideas regarding how feedback could be organized within the clinical workplace to promote feedback processes. The feedback encounter should be organized to allow follow-up of the feedback, i.e., working on required learning and performance goals at the next occasion. The educational programs should design feedback processes by appropriately planning subsequent tasks and activities. More insight is needed in designing a full-loop feedback process, in which specific attention is needed in effective feedforward practices.

**Supplementary Information:**

The online version contains supplementary material available at 10.1186/s12909-024-05439-6.

## Background

The design of effective feedback processes in higher education has been important for educators and researchers and has prompted numerous publications discussing potential mechanisms, theoretical frameworks, and best practice examples over the past few decades. Initially, research on feedback primarily focused more on teachers and feedback delivery, and students were depicted as passive feedback recipients [1–3]. The feedback conversation has recently evolved to a more dynamic emphasis on interaction, sense-making, outcomes in actions, and engagement with learners [2]. This shift aligns with utilizing the feedback process as a form of social interaction or dialogue to enhance performance [4]. Henderson et al. (2019) defined feedback processes as "where the learner makes sense of performance-relevant information to promote their learning." (p. 17). When a student grasps the information concerning their performance in connection to the desired learning outcome and subsequently takes suitable action, a feedback loop is closed so the process can be regarded as successful [5, 6].

Hattie and Timperley (2007) proposed a comprehensive perspective on feedback, the so-called feedback loop, to answer three key questions: “Where am I going? “How am I going?” and “Where to next?” [7]. Each question represents a key dimension of the feedback loop. The first is the feed-up, which consists of setting learning goals and sharing clear objectives of learners' performance expectations. While the concept of the feed-up might not be consistently included in the literature, it is considered to be related to principles of effective feedback and goal setting within educational contexts [7, 8]. Goal setting allows students to focus on tasks and learning, and teachers to have clear intended learning outcomes to enable the design of aligned activities and tasks in which feedback processes can be embedded [9]. Teachers can improve the feed-up dimension by proposing clear, challenging, but achievable goals [7]. The second dimension of the feedback loop focuses on feedback and aims to answer the second question by obtaining information about students' current performance. Different teaching and assessment activities can be used to obtain feedback information, and it can be provided by a teacher or tutor, a peer, oneself, a patient, or another coworker. The last dimension of the feedback loop is the feedforward, which is specifically associated with using feedback to improve performance or change behaviors [10]. Feedforward is crucial in closing the loop because it refers to those specific actions students must take to reduce the gap between current and desired performance [7].

From a sociocultural perspective, feedback processes involve a social practice consisting of intricate relationships within a learning context [11]. The main feature of this approach is that students learn from feedback only when the feedback encounter includes generating, making sense of, and acting upon the information given [11]. In the context of workplace-based learning (WBL), actionable feedback plays a crucial role in enabling learners to leverage specific feedback to enhance their performance, skills, and conceptual understandings. The WBL environment provides students with a valuable opportunity to gain hands-on experience in authentic clinical settings, in which students work more independently on real-world tasks, allowing them to develop and exhibit their competencies [3]. However, WBL settings are characterized by their unpredictable nature, which can either promote self-directed learning or present challenges in offering structured learning opportunities for students [12]. Consequently, designing purposive feedback opportunities within WBL settings is a significant challenge for clinical teachers and faculty.

In undergraduate clinical education, feedback opportunities are often constrained due to the emphasis on clinical work and the absence of dedicated time for teaching [13]. Students are expected to perform autonomously under supervision, ideally achieved by giving them space to practice progressively and providing continuous instances of constructive feedback [14]. However, the hierarchy often present in clinical settings places undergraduate students in a dependent position, below residents and specialists [15]. Undergraduate or junior students may have different approaches to receiving and using feedback. If their priority is meeting the minimum standards given pass-fail consequences and acting merely as feedback recipients, other incentives may be needed to engage with the feedback processes because they will need more learning support [16, 17]. Adequate supervision and feedback have been recognized as vital educational support in encouraging students to adopt a constructive learning approach [18]. Given that productive feedback processes rely on observed information regarding a student's performance, it is imperative to establish structured teaching and learning feedback activities within undergraduate WBL settings.

Despite the extensive research on feedback, a significant proportion of published studies involve residents or postgraduate students [19, 20]. Recent reviews focusing on feedback interventions within medical education have clearly distinguished between undergraduate medical students and residents or fellows [21]. To gain a comprehensive understanding of initiatives related to actionable feedback in the WBL environment for undergraduate health professions, a scoping review of the existing literature could provide insight into how feedback processes are designed in that context. Accordingly, the present scoping review aims to answer the following research question: How are the feedback processes designed in the undergraduate health professions' workplace-based learning environments?

## Methods

A scoping review was conducted using the five-step methodological framework proposed by Arksey and O'Malley (2005) [22], intertwined with the PRISMA checklist extension for scoping reviews to provide reporting guidance for this specific type of knowledge synthesis [23]. Scoping reviews allow us to study the literature without restricting the methodological quality of the studies found, systematically and comprehensively map the literature, and identify gaps [24]. Furthermore, a scoping review was used because this topic is not suitable for a systematic review due to the varied approaches described and the large difference in the methodologies used [21].

### Search strategy

With the collaboration of a medical librarian, the authors used the research question to guide the search strategy. An initial meeting was held to define keywords and search resources. The proposed search strategy was reviewed by the research team, and then the study selection was conducted in two steps:

1. An online database search included Medline/PubMed, Web of Science, CINAHL, Cochrane Library, Embase, ERIC, and PsycINFO.
2. A directed search of ten relevant journals in the health sciences education field (Academic Medicine, Medical Education, Advances in Health Sciences Education, Medical Teacher, Teaching and Learning in Medicine, Journal of Surgical Education, BMC Medical Education, Medical Education Online, Perspectives on Medical Education and The Clinical Teacher) was performed.

The research team conducted a pilot or initial search before the full search to identify if the topic was susceptible to a scoping review. The full search was conducted in November 2022. One team member (MO) identified the papers in the databases. JF searched in the selected journals. Authors included studies written in English due to feasibility issues, with no time span limitation. After eliminating duplicates, two research team members (JF and IV) independently reviewed all the titles and abstracts using the exclusion and inclusion criteria described in Table 2 and with the support of the screening application StArT [25]. A third team member (AR) reviewed the titles and abstracts when the first two disagreed. The reviewer team met again at a midpoint and final stage to discuss the challenges related to study selection. Articles included for full-text review were exported to Mendeley. JF independently screened all full-text papers, and AR verified 10% for inclusion. The authors did not analyze study quality or risk of bias during study selection, which is consistent with conducting a scoping review.

The analysis of the results incorporated a descriptive summary and a thematic analysis, which was carried out to clarify and give consistency to the results' reporting [22, 24, 26]. Quantitative data were analyzed to report the characteristics of the studies, populations, settings, methods, and outcomes. Qualitative data were labeled, coded, and categorized into themes by three team members (JF, SH, and DS). The feedback loop framework with a sociocultural perspective was used as the theoretical framework to analyze the results.

The keywords used for the search strategies were as follows:

Clinical clerkship; feedback; formative feedback; health professions; undergraduate medical education; workplace.

Definitions of the keywords used for the present review are available in Appendix 1.

As an example, we included the search strategy that we used in the Medline/PubMed database when conducting the full search:

("Formative Feedback"[Mesh] OR feedback) AND ("Workplace"[Mesh] OR workplace OR "Clinical Clerkship"[Mesh] OR clerkship) AND (("Education, Medical, Undergraduate"[Mesh] OR undergraduate health profession*) OR (learner* medical education)).

### Inclusion and exclusion criteria

The following inclusion and exclusion criteria were used (Table 1):

**Table 1:** Exclusion and inclusion criteria

	Exclusion	Inclusion
Population	Residents, postgraduate students, fellows, attendings, staff, house officers, and house staff	Undergraduate students of any health profession
Context	Campus-based learning, simulation lab	Workplace-based learning (inpatient or outpatient settings)
Intervention	Pre-clinical course/intervention	Any feedback practice described within clinical education in the WBL setting
Language	Other language than English	English written studies

### Data extraction

The research group developed a data-charting form to organize the information obtained from the studies. The process was iterative, as the data chart was continuously reviewed and improved as necessary. In addition, following Levac et al.'s recommendation (2010), the three members involved in the charting process (JF, LI, and IV) independently reviewed the first five selected studies to determine whether the data extraction was consistent with the objectives of this scoping review and to ensure consistency. Then, the team met using web-conferencing software (Zoom; CA, USA) to review the results and adjust any details in the chart. The same three members extracted data independently from all the selected studies, considering two members reviewing each paper [26]. A third team member was consulted if any conflict occurred when extracting data. The data chart identified demographic patterns and facilitated the data synthesis. To organize data, we used a shared Excel spreadsheet, considering the following headings: title, author(s), year of publication, journal/source, country/origin, aim of the study, research question (if any), population/sample size, participants, discipline, setting, methodology, study design, data collection, data analysis, intervention, outcomes, outcomes measure, key findings, and relation of findings to research question.

Additionally, all the included papers were uploaded to AtlasTi v19 to facilitate the qualitative analysis. Three team members (JF, SH, and DS) independently coded the first six papers to create a list of codes to ensure consistency and rigor. The group met several times to discuss and refine the list of codes. Then, one member of the team (JF) used the code list to code all the rest of the papers. Once all papers were coded, the team organized codes into descriptive themes aligned with the research question.

Preliminary results were shared with a number of stakeholders (six clinical teachers, ten students, six medical educators) to elicit their opinions as an opportunity to build on the evidence and offer a greater level of meaning, content expertise, and perspective to the preliminary findings [26]. No quality appraisal of the studies is considered for this scoping review, which aligns with the frameworks for guiding scoping reviews [27].

The datasets analyzed during the current study are available from the corresponding author upon request.

## Results

**Fig. 1:**
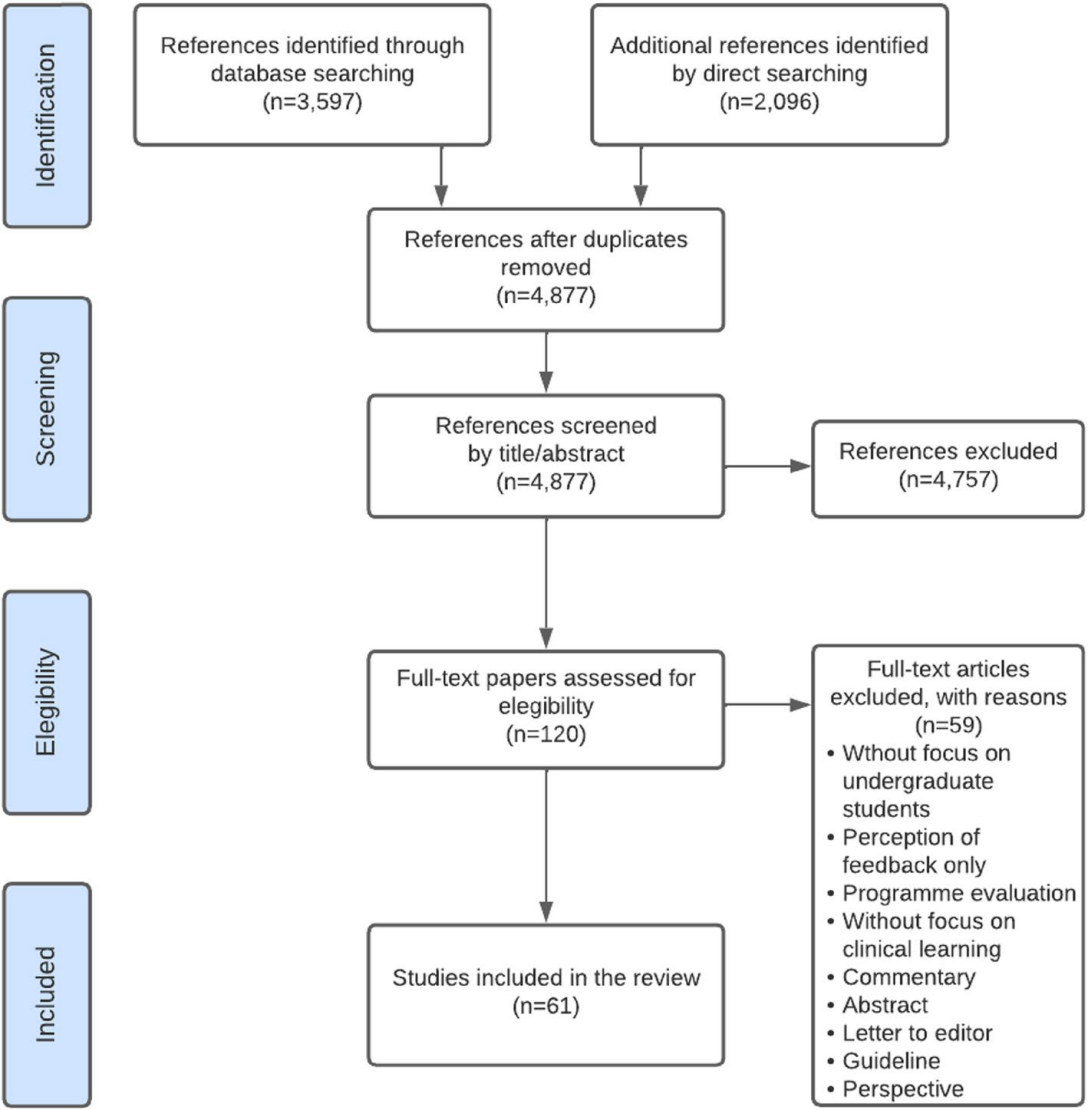
PRISMA flow diagram for included studies, incorporating records identified through the database and direct searching

A database search resulted in 3,597 papers, and the directed search of the most relevant journals in the health sciences education field yielded 2,096 titles. An example of the results of one database is available in Appendix 2. Of the titles obtained, 816 duplicates were eliminated, and the team reviewed the titles and abstracts of 4,877 papers. Of these, 120 were selected for full-text review. Finally, 61 papers were included in this scoping review (Fig. 1), as listed in Table 2.

**Table 2:** Papers included in the scoping review. N/A: not available information, i.e., the paper does not explicitly state which discipline it refers to

Author	Title	Year	Publication	Type of document	Country/Origin	Aim of the study	Discipline
Adamson, E; Kinga, L; Foy, L; McLeodb, M; Traynor, J; Watson, W; Gray, W	Feedback in clinical practice: Enhancing the students’ experience through action research	2018	Nurse Education in Practice	Original paper	UK	To raise awareness, and provide support and training for mentors within clinical practice in relation to the provision of explicit and appropriate feedback to students on their practice To enhance student nurse understanding of the many forms that feedback within placements might take and how to apply this to their practice	Nursing
Al-Mously, N; Nabil, N; Al-Babtain, S; Fouad Abbas, M	Undergraduate medical students' perceptions on the quality of feedback received during clinical rotations	2014	Medical Teacher	Original paper	Saudi Arabia	To report undergraduate medical students' evaluation of the frequency and the quality of feedback received on their clinical performance during their clerkships	Medicine
Bates, J; Konkin, J; Suddards, C; Dobson, S; Pratt, D	Student perceptions of assessment and feedback in longitudinal integrated clerkships	2013	Medical Education	Original paper	Canada	To elucidate how the learning environment and the student-preceptor relationship influence student experiences of being assessed	Medicine
Bennett, A; Goldenhar, L; Stanford, K	Utilization of a Formative Evaluation Card in a Psychiatry Clerkship	2006	Academic Psychiatry	Original paper	USA	To discuss how formative feedback to medical students during their clinical rotations facilitates their successfully meeting the rotation’s educational objectives	Medicine
Bing-You, R; Hayes, V; Palka, T; Ford, M; Trowbridge, R	The Art (and Artifice) of Seeking Feedback: Clerkship Students’ Approaches to Asking for Feedback	2018	Academic Medicine	Original paper	USA	To explore feedback-seeking behaviours of medical students within clerkship	Medicine
Bing-You, R; Hayes, V; Varaklis, K; Trowbridge, R; Kemp, H; McKelvy, D	Feedback for Learners in Medical Education: What is Known? A Scoping Review	2017	Academic Medicine	Review	USA	To explore what is known about feedback as a means of learner improvement in the field of medical education	N/A
Bing-You, R; Varaklis, K; Trowbridge, R; Kemp, H; McKelvy, D	The Feedback Tango: An Integrative Review and Analysis of the Content of the Teacher-Learner Feedback Exchange	2018	Academic Medicine	Review	USA	To conduct an integrative review and analysis of the literature on the content of feedback to learners in medical education	N/A
Bok, H; Jaarsma, D; Spruijt, A; van Beukelen, P; van der Vleuten, C; Teunissen, P	Feedback-giving behaviour in performance evaluations during clinical clerkships	2016	Medical Teacher	Original paper	Netherlands	To investigate teachers’ use of mini-CEX in performance evaluations to provide narrative feedback in undergraduate clinical training	Veterinary medicine
Bok, Harold G J; Teunissen, Pim W; Spruijt, Annemarie; Fokkema, Joanne P I; Van Beukelen, Peter; Jaarsma, Debbie A D C; Van Der Vleuten, Cees P M	Clarifying students' feedback-seeking behaviour in clinical clerkships	2013	Medical Education	Original paper	Netherlands	To explore students’ feedback-seeking behaviours in the clinical workplace	Veterinary medicine
Calleja, P.; Harvey, T.; Fox, A.; Carmichael, M	Feedback and clinical practice improvement: A tool to assist workplace supervisors and students	2016	Nurse Education in Practice	2016	Australia	To evaluate the implementation of the feedback and clinical practice improvement tool and processes, and identify common conduits and barriers identified as impacting on learning	Nursing; Radiation Therapy
Carey, E; Wu, C; Hur, E; Hasday, S; Rosculet, N; Kemp, M; Weir, S; Ryszawa, S; Sandhu, G; Hughes, D; Reddy, R	Evaluation of Feedback Systems for the Third-Year Surgical Clerkship	2017	Journal of Surgical Education	2017	USA	To compare faculty-to-student feedback rates from 2 different data sets: direct observation cards and end-of-clerkship questionnaires	Medicine
Crommelinck, M; Anseel, F	Understanding and encouraging feedback-seeking behaviour: a literature review	2013	Medical Education	Review	Belgium	To review the literature on feedback-seeking behaviour using a self-motives framework and to provide practical recommendations for medical educators on how to encourage feedback-seeking behaviour	Medicine
Daelmans, H; Overmeer, R; Van Der Hem-Stokroos, H; Scherpbier, A; Stehouwer, C; Van Der Vleuten, C;	In-training assessment: qualitative study of effects on supervision and feedback in an undergraduate clinical rotation	2006	Medical Education	Original paper	Netherlands	To investigate an in-training assessment (ITA) programme in action and to explore its effects on supervision and feedback	Medicine
DeWitt, D; Carline, J; Paauw, D; Pangaro, L	Pilot study of a 'RIME'-based tool for giving feedback in a multi-specialty longitudinal clerkship	2008	Medical Education	Original paper	UK	To develop and pilot a RIME-based feedback tool	Medicine
Dolan, B; O´Brien, C; Green, M	Including Entrustment Language in an Assessment Form May Improve Constructive Feedback for Student Clinical Skills	2017	Medical Science Educator	Original paper	USA	To determine if faculty provide more constructive and specific feedback to learners after the inclusion of an assessment item including entrustment language in standard assessment forms	Medicine
Duijn, C; Welink, L; Mandoki, M; ten Cate, O; Kremer, W; Bok, H	Am I ready for it? Students’ perceptions of meaningful feedback on entrustable professional activities	2017	Perspectives on Medical Education	Original paper	Netherlands and Hungary	To illustrate what students’ perceptions are of meaningful feedback viewed as conducive in preparing for performing EPA unsupervised	Medicine and Veterinary
Elnicki, D Michael; Zalenski, Dianne	Integrating medical students' goals, self-assessment and preceptor feedback in an ambulatory clerkship	2013	Teaching and Learning in Medicine	Original paper	USA	To determine whether student self-assessment and preceptor feedback correlate with course outcomes and whether preceptor feedback informs student self-assessment	Medicine
Embo, M; Driessen, E; Valcke, M; van der Vleuten, C	Assessment and feedback to facilitate self-directed learning in clinical practice of midwifery students	2010	Medical Teacher	Original paper	Belgium	To explore students’ perceptions about a newly introduced integrated feedback and assessment instrument to support self- directed learning in clinical practice	Midwifery
Eva, K; Armson, H; Holmboe, E; Lockyer, J	Factors influencing responsiveness to feedback: on the interplay between fear, confidence, and reasoning processes	2016	Advances in Health Sciences Education	Original paper	Canada, UK,The Netherlands, USA, Australia	To explore factors which aid or hinder receptivity to feedback	Medicine and Midwifery
Farrell, L; Bourgeois-Law, G; Ajjawi, R; Regehr, G	An autoethnographic exploration of the use of goal-oriented feedback to enhance brief clinical teaching encounters	2017	Advances in Health Sciences Education	Original paper	Canada and Australia	To explore the use of goal-oriented feedback in brief encounters with learners	Medicine
Fernando, N; Cleland, J; McKenzie, H; Cassar, K	Identifying the factors that determine feedback given to undergraduate medical students following formative mini-CEX assessments	2008	Medical Education	Original paper	UK	To examine the factors that determine provision of feedback to students follow- ing mini-clinical evaluation exercise (mini-CEX) assessments	Medicine
Garino, Alexandria	Ready, willing and able: a model to explain successful use of feedback	2020	Advances in Health Sciences Education	Original paper	USA	To examine the behaviors and learner characteristics that contribute to successful use of feedback. To explore how medicine and physician assistant students engage with feedback and if motivational goals could explain learner differences	Medicine and Physician Assistants
Garner, Matthew S.; Gusberg, Richard J.; Kim, Anthony W	The Positive Effect of Immediate Feedback on Medical Student Education During the Surgical Clerkship	2014	Surgical Education	Original paper	USA	To determine if providing feedback Immediately following a meaningful interaction with surgical faculty would be advantageous to the medical student learning in their third-year clerkship	Medicine
Haffling, A; Beckman, A; Edgren, G	Structured feedback to undergraduate medical students: 3 years’ experience of an assessment tool	2011	Medical Teacher	Original paper	Sweden	To investigate the outcome of long-term use of an assessment tool	Medicine
Harrison, C. J., Könings, K. D., Dannefer, E. F., Schuwirth, L. W., Wass, V., & van der Vleuten, C. P	Factors inffuencing students’ receptivity to formative feedback emerging from different assessment cultures	2016	Perspectives on Medical Education	Original paper	USA and Canada	To better inform various educationally relevant activities including undergraduate and postgraduate training prac- tices, remediation, continuing education, and knowledge translation	Medicine and Midwifery
Harrison, C; Könings, K; Schuwirth, L; Wass, V; van der Vleuten, C	Changing the culture of assessment: the dominance of the summative assessment paradigm	2017	BMC Medical Education	Original paper	UK	To explore an institution’s readiness to adopt initial changes which would help an organisation move towards an assessment for learning culture	Medicine
Harvey, P; Radomski, N; O’Connor, D	Written feedback and continuity of learning in a geographically distributed medical education program	2013	Medical Teacher	Original paper	Australia	To investigate how clinical supervisors construct performance orientated written feedback and learning goals for medical students in a geographically distributed medical education (GDME) programme	Medicine
Hochberg, M; Berman, R; Ogilvie, J; Yingling, S; Lee, S; Pusic, M; Pachter, HL	Midclerkship feedback in the surgical clerkship: the "Professionalism, Reporting, Interpreting, Managing, Educating, and Procedural Skills" application utilizing learner self-assessment	2017	The American Journal of Surgery	Original paper	USA	To determine the feasibility of collecting and comparing student self-assessment with that of their preceptors using an iPad application	Medicine
Holmboe, E; Yepes, M; Williams, F; Huot, S	Feedback and the mini clinical evaluation exercise	2004	Journal of General Internal Medicine	Original paper	USA	To examine how often faculty provided recommendations and used interactive techniques when providing feedback as part of a miniCEX	Medicine
Johnson, C; Keating, J; Molloy, E	Psychological safety in feedback: What does it look like and how can educators work with learners to foster it?	2020	Medical Education	Original paper	Australia	To explore psychological safety in workplace feedback and how can educators work with learners to foster it	Medicine
Joshi, A; Generalla, J; Thompson, B; Haidet, P	Facilitating the Feedback Process on a Clinical Clerkship Using a Smartphone Application	2017	Academic Psychiatry	Original paper	USA	To evaluate the effects of a smartphone-triggered method of feedback delivery on students’ perceptions of the feedback process	Medicine
Kiger, Michelle E; Riley, Caylin; Stolfi, Adrienne; Morrison, Stephanie; Burke, Ann; Lockspeiser, Tai	Use of Individualized Learning Plans to Facilitate Feedback Among Medical Students	2020	Teaching and Learning in Medicine	Original paper	USA	To determine whether having medical students share their ILPs with their attending physicians or supervising residents affected (1) the quality of the feedback they received, as measured by a feedback scoring rubric; (2) the degree of correlation between the content of the learners’ ILPs and the content of their feedback; and (3) learner perceptions of their feedback	Medicine
Kogan JR; Shea JA	Implementing feedback cards in core clerkships	2008	Medical Education	Original paper	USA	To determine the feasibility of a cross-clerkship feedback encounter card system, describe the content of feedback requested and received during the core clerkships, and examine student satisfaction with the feedback card system	Medicine
Lefroy, J; Walters, B; Molyneux, A; Smithson, S	Can learning from workplace feedback be enhanced by reflective writing? A realist evaluation in UK undergraduate medical education	2021	Education for Primary Care	Original paper	UK	To explain what it is about the ‘Learning from feedback’ system which is working or not working for students and why	Medicine
Long, S; Rodriguez, C; St-Onge, C; Tellier, PP; Torabi, N; Young, M	Factors affecting perceived credibility of assessment in medical education: A scoping review	2022	Advances in Health Sciences Education	Scoping review	Canada	To understand how to support learner engagement with assessment-generated feedback by documenting assessment practices perceived as credible (or not) by learners	Medical Education
McGinness, Hannah T.; Caldwell, Patrina H. Y.; Gunasekera, Hasantha; Scott, Karen M	"Every Human Interaction Requires a Bit of Give and Take": Medical Students Approaches to Pursuing Feedback in the Clinical Setting	2022	Teaching and Learning in Medicine	Original paper	Australia	To explore influences on medical student feedback behavior during clinical attachments	Medicine
Noble, C; Billett, S; Armit, L; Collier, L; Hilder, J; Sly, C; Molloy, E	``It's yours to take{''}: generating learner feedback literacy in the workplace	2020	Advances in Health Sciences Education	Original paper	Australia	To problematise student feedback literacy in the healthcare setting, from the learner’s perspective	Nursing, Social Work, and Medicine
Ogburn, T; Espey, E	The R-I-M-E method for evaluation of medical students on an obstetrics and gynecology clerkship	2018	American Journal of Obstetrics & Gynecology	Original paper	USA	To implement and assess the R-I-M-E (Reporter, Interpreter, Manager, Educator) system as a method for evaluation for medical students during the obstetrics and gynecology core clerkship	Medicine
Ossenberg, C; Henderson, A; Mitchell, M	What attributes guide best practice for effective feedback? A scoping review	2019	Advances in Health Sciences Education	Review	Australia	To identify the attributes that contribute to effective feedback within a dialogic lens and. To better understand whether they align with the contemporary discourse of dialogic feedback. To explore if the attributes can promote student engagement in feedback in the workplace based learning environment	Allied Health, Business, Education, Medicine, Nursing and Psychology
Ozuah PO; Reznik M; Greenberg L	Improving a medical student feedback with a clinical encounter card	2007	Ambulatory Pediatrics	Original paper	USA	To determine whether the use of the Clinical Encounter Card (CEC) would increase medical students´perception of the feedback they recieved	Medicine
Parkes, J; Abercrombie, S; McCarty, T	Feedback sandwiches affect perceptions but not performance	2013	Advances in Health Sciences Education	Original paper	USA	To explore students’ perceptions of the impact of the feedback sandwich technique on their performance	Medicine
Paukert, Judy L; Richards, Melanie L; Olney, Cynthia	An encounter card system for increasing feedback to students	2002	The American Journal of Surgery	Original paper	USA	To assess the effectiveness and initial implementation of this method for increasing feedback received by third-year medical students from faculty, fellows, and residents during a 12-week surgery clerkship	Medicine
Rassos, James; Melvin, Lindsay J.; Panisko, Daniel; Kulasegaram, Kulamakan; Kuper, Ayelet	Faculty and Trainee Perspectives of Feedback in Internal Medicine: the Oral Case Presentation as a Model	2019	Journal of General Internal Medicine	Original paper	Canada	To explore how Internal Medicine clinical supervisors and trainees perceive feedback within the context of the case presentation	Medicine
Rizan, C; Elsey, C; Lemon, T; Grant, A; Monrouxe, L	Feedback in action within bedside teaching encounters: A video ethnographic study	2014	Medical Education	Original paper	UK	To explore the interactional patterns and correction modalities utilised in feedback sequences between doctors and students within general practice-based bedside teaching encounters (BTEs)	Medicine
Robertson, AC; Fowler, LC	Medical Student Perceptions of Learner-Initiated Feedback Using a Mobile Web Application	2017	Journal of Medical Education and Curricular Development	Original paper	USA	To identify perceptions of medical students about faculty feedback and soliciting faculty feedback using a mobile device and Web-based application	Medicine
Scheidt, Peter C.; And Others	Evaluation of System Providing Feedback to Students on Videotaped Patient Encounters	1986	Journal of Medical Education	Original paper	USA	To determine wther students who recieve critiques of the videotapes of actual patient encounters from their preceptors perform subsequent interviews and examinations more skillfully than students who recieve only a self-guided crtitique or no critique at all	Medicine
Schopper, H; Rosenbaum, M; Axelson, R	“I wish someone watched me interview:” medical student insight into observation and feedback as a method for teaching communication skills during the clinical years	2016	BMC Medical Education	Original paper	USA	To explore student perspectives regarding their experiences with clinical observation and feedback on communication skills	Medicine
Soemantri D.; Dodds A.; Mccoll G	Examining the nature of feedback within the Mini Clinical Evaluation Exercise (Mini-CEX): an analysis of 1427 Mini-CEX assessment forms	2018	GMS Journal of Medical Education	Original paper	Australia	To examine the written feedback provided on the Mini-CEX form to determine its usefulness as a learning tool for students	Medicine
Sokol-Hessner, L; Shea, J; Kogan, J	The open-ended comment space for action plans on core clerkship students’ encounter cards: What gets written?	2010	Academic Medicine	Original paper	USA	To characterize written comments on encounter cards with space designated for an action plan	Medicine
Sox, CM; Dell, M; Phillipi, CA; Cabral, HJ; Vargas, G; Lewin, LO	Feedback on oral presentations during pediatric clerkships: a randomized controlled trial	2014	Pediatrics	Original paper	USA	To measure the effects of participating in structured oral presentation evaluation sessions early in pediatric clerkships on students’ subsequent presentations	Medicine
Spickard, A; Gigante, J; Stein, G; Denny, J	Automatic capture of student notes to augment mentor feedback and student performance on patient write-ups	2008	Journal of General Internal Medicine	Original paper	USA	To determine whether the integration of an automated electronic clinical portfolio into clinical clerkships can improve the quality of feedback given to students on their patient write-ups and the quality of students’ write-ups	Medicine
Spooner, M; Duane, C; Uygur, J; Smyth, E; Marron, B; Murphy, PJ; Pawlikowska, T	Self -regulatory learning theory as a lens on how undergraduate and postgraduate learners respond to feedback: A BEME scoping review: BEME Guide No. 66	2022	Medical Teacher	Scoping review	Ireland	To map what is known of how learners interact with feedback, to better understand how feedback affects learning strategies, and to explore enhancing and inhibiting factors	Medical Education
Suhoyo Y.; Schonrock-Adema J.; Emilia O.; Kuks J.B.M.; Cohen-Schotanus J	Clinical workplace learning: perceived learning value of individual and group feedback in a collectivistic culture	2014	Medical Teacher	Original paper	Indonesia	To investigate the perceived learning value and characteristics of individual and group feedback in a collectivistic culture	Medicine
Suhoyo, Y; Van Hell, E; Kerdijk, W; Emilia, O; Schönrock-Adema, J; Kuks, J; Cohen-Schotanus, J	Influence of feedback characteristics on perceived learning value of feedback in clerkships: Does culture matter?	2017	BMC Medical Education	Original paper	Indonesia	To validate the influence of five feedback characteristics on students’ perceived learning value of feedback in an Indonesian clerkship context	Medicine
Tai, J; Canny, B; Haines, T; Molloy, E	The role of peer-assisted learning in building evaluative judgement: opportunities in clinical medical education	2016	Advances in Health Sciences Education	Original paper	Australia	To explore the contribution of peer-assisted learning (PAL) in the development of evaluative judgement capacity; the ability to understand work quality and apply those standards to appraising performance	Medicine
Torre, D; Simpson, D; Sebastian, J; Elnicki, D	Learning/Feedback Activities and High-Quality Teaching: Perceptions of Third-Year Medical Students during an Inpatient Rotation	2005	Academic Medicine	Original paper	USA	To identify specific learning activities (and teaching methods) that students associate with high-quality teaching in the inpatient setting	Medicine
Urquhart, L. M.; Ker, J. S.; Rees, C. E	Exploring the Influence of Context on Feedback at Medical School: A Video-Ethnography Study	2018	Advances in Health Sciences Education	Original paper	UK	To explore gaps in the published literature about how context influences feedback processes	Medicine
van de Ridder, M Stokking, M; McGaghie, W; ten Cate, O	What is feedback in clinical education?	2008	Medical Education	Review	The Netherlands and USA	To propose a consensual, research-based, operational definition of feedback in clinical education	N/A
van de Ridder, M; McGaghie, W; Stokking, K; ten Cate, O	Variables that affect the process and outcome of feedback, relevant for medical training: A meta-review	2015	Medical Education	Review	The Netherlands and USA	To determine which variables influence the process and outcomes of feedback in settings relevant to medical education	N/A
Watling, C; Driessen, E; van der Vleuten, C; Lingard, L	Learning culture and feedback: an international study of medical athletes and musicians	2014	Medical Education	Original paper	Canada, USA, and the Netherlands	To distinguish the elements of the response to feedback that are determined by the individual learner from those determined by the learning culture, and to understand how these elements interact in order to make recommendations for improving feedback in medical education	Medicine
Watling, C; Driessen, E; van der Vleuten, C; Vanstone, M; Lingard, L	Beyond individualism: Professional culture and its influence on feedback	2013	Medical Education	Original paper	Canada	To explore how feedback is handled within different professional cultures, and how the characteristics and values of a profession shape learners’ responses to feedback	Medicine, Music, and Teacher Training

The selected studies were published between 1986 and 2022, and seventy-five percent (46) were published during the last decade. Of all the articles included in this review, 13% (8) were literature reviews: one integrative review [28] and four scoping reviews [29–32]. Finally, fifty-three (87%) original or empirical papers were included (i.e., studies that answered a research question or achieved a research purpose through qualitative or quantitative methodologies) [15, 33–85].

Table 2 summarizes the papers included in the present scoping review, and Table 3 describes the characteristics of the included studies.

**Table 3:** Characteristics of included studies

Continent of publication	Number	%
North America and Canada	33	53,2%
Europe	17	27,4%
Australia	8	12,9%
Asia	3	4,8%
**Methodology**		
Qualitative	26	42%
Quantitative	25	40%
Reviews	8	13%
Mixed methods	2	3%
**Discipline**^a^		
Medicine	42	79%
Veterinary Medicine	2	4%
Medicine and Midwifery	2	4%
Three or more disciplines	2	4%
Nursing	1	2%
Midwifery	1	2%
Medicine and Physician Assistance	1	2%
Medicine and Veterinary Medicine	1	2%
Nursing; Radiation Therapy	1	2%

^a^Only including original studies

The thematic analysis resulted in two themes: (1) the organization of feedback processes in WBL settings, and (2) sociocultural factors influencing the organization of feedback processes. Table 4 gives a summary of the themes and subthemes.

**Table 4:** Themes and subthemes identified in the qualitative analysis

1. Organization of feedback processes in WBL settings
1.1. Setting learning goals (i.e., feed-up dimension)
1.2. Feedback strategies within the WBL environment. (i.e., feedback dimension)
1.3. Organization of follow-up feedback and action plans (i.e., feedforward dimension)
2. Sociocultural factors influencing the organization of feedback processes
1.1. Clinical learning culture
1.2. Relationships
1.3. Students as active agents in the feedback processes

## Organization of feedback processes in WBL settings.

### Setting learning goals (i.e., feed-up dimension)

Feedback that focuses on students' learning needs and is based on known performance standards enhances student response and setting learning goals [30]. Discussing goals and agreements before starting clinical practice enhances students' feedback-seeking behavior [39] and responsiveness to feedback [83]. Farrell et al. (2017) found that teacher-learner co-constructed learning goals enhance feedback interactions and help establish educational alliances, improving the learning experience [50]. However, Kiger (2020) found that sharing individualized learning plans with teachers aligned feedback with learning goals but did not improve students' perceived use of feedback [64]

Two papers of this set pointed out the importance of goal-oriented feedback, a dynamic process that depends on discussion of goal setting between teachers and students [50] and influences how individuals experience, approach, and respond to upcoming learning activities [34]. Goal-oriented feedback should be embedded in the learning experience of the clinical workplace, as it can enhance students' engagement in safe feedback dialogues [50]. Ideally, each feedback encounter in the WBL context should conclude, in addition to setting a plan of action to achieve the desired goal, with a reflection on the next goal [50].

### Feedback strategies within the WBL environment. (i.e., feedback dimension)

In undergraduate WBL environments, there are several tasks and feedback opportunities organized in the undergraduate clinical workplace that can enable feedback processes:

***Questions*** from clinical teachers to students are a feedback strategy [74]. There are different types of questions that the teacher can use, either to clarify concepts, to reach the correct answer, or to facilitate self-correction [74]. Usually, questions can be used in conjunction with other communication strategies, such as pauses, which enable self-correction by the student [74]. Students can also ask questions to obtain feedback on their performance [54]. However, question-and-answer as a feedback strategy usually provides information on either correct or incorrect answers and fewer suggestions for improvement, rendering it less constructive as a feedback strategy [82].

***Direct observation*** of performance by default is needed to be able to provide information to be used as input in the feedback process [33, 46, 49, 86]. In the process of observation, teachers can include clarification of objectives (i.e., feed-up dimension) and suggestions for an action plan (i.e., feedforward) [50]. Accordingly, Schopper et al. (2016) showed that students valued being observed while interviewing patients, as they received feedback that helped them become more efficient and effective as interviewers and communicators [33]. Moreover, it is widely described that direct observation improves feedback credibility [33, 40, 84]. Ideally, observation should be deliberate [33, 83], informal or spontaneous [33], conducted by a (clinical) expert [46, 86], provided immediately after the observation, and clinical teacher if possible, should schedule or be alert on follow-up observations to promote closing the gap between current and desired performance [46].

***Workplace-based assessments*** (WBAs), by definition, entail direct observation of performance during authentic task demonstration [39, 46, 56, 87]. WBAs can significantly impact behavioral change in medical students [55]. Organizing and designing formative WBAs and embedding these in a feedback dialogue is essential for effective learning [31].

Summative organization of WBAs is a well described barrier for feedback uptake in the clinical workplace [35, 46]. If feedback is perceived as summative, or organized as a pass-fail decision, students may be less inclined to use the feedback for future learning [52]. According to Schopper et al. (2016), using a scale within a WBA makes students shift their focus during the clinical interaction and see it as an assessment with consequences [33]. Harrison et al. (2016) pointed out that an environment that only contains assessments with a summative purpose will not lead to a culture of learning and improving performance [56]. The recommendation is to separate the formative and summative WBAs, as feedback in summative instances is often not recognized as a learning opportunity or an instance to seek feedback [54]. In terms of the design, an organizational format is needed to clarify to students how formative assessments can promote learning from feedback [56]. Harrison et al. (2016) identified that enabling students to have more control over their assessments, designing authentic assessments, and facilitating long-term mentoring could improve receptivity to formative assessment feedback [56].

Multiple WBA instruments and systems are reported in the literature. Sox et al. (2014) used a detailed evaluation form to help students improve their clinical case presentation skills. They found that feedback on oral presentations provided by supervisors using a detailed evaluation form improved clerkship students’ oral presentation skills [78]. Daelmans et al. (2006) suggested that a formal in-training assessment programme composed by 19 assessments that provided structured feedback, could promote observation and verbal feedback opportunities through frequent assessments [43]. However, in this setting, limited student-staff interactions still hindered feedback follow-up [43]. Designing frequent WBA improves feedback credibility [28]. Long et al. (2021) emphasized that students' responsiveness to assessment feedback hinges on its perceived credibility, underlining the importance of credibility for students to effectively engage and improve their performance [31].

The mini-CEX is one of the most widely described WBA instruments in the literature. Students perceive that the mini-CEX allows them to be observed and encourages the development of interviewing skills [33]. The mini-CEX can provide feedback that improves students' clinical skills [58, 60], as it incorporates a structure for discussing the student's strengths and weaknesses and the design of a written action plan [39, 80]. When mini-CEXs are incorporated as part of a system of WBA, such as programmatic assessment, students feel confident in seeking feedback after observation, and being systematic allows for follow-up [39]. Students suggested separating grading from observation and using the mini-CEX in more informal situations [33].

Clinical encounter cards allow students to receive weekly feedback and make them request more feedback as the clerkship progresses [65]. Moreover, encounter cards stimulate that feedback is given by supervisors, and students are more satisfied with the feedback process [72]. With encounter card feedback, students are responsible for asking a supervisor for feedback before a clinical encounter, and supervisors give students written and verbal comments about their performance after the encounter [42, 72]. Encounter cards enhance the use of feedback and add approximately one minute to the length of the clinical encounter, so they are well accepted by students and supervisors [72]. Bennett (2006) identified that Instant Feedback Cards (IFC) facilitated mid-rotation feedback [38]. Feedback encounter card comments must be discussed between students and supervisors; otherwise, students may perceive it as impersonal, static, formulaic, and incomplete [59].

***Self-assessments*** can change students' feedback orientation, transforming them into coproducers of learning [68]. Self-assessments promote the feedback process [68]. Some articles emphasize the importance of organizing self-assessments before receiving feedback from supervisors, for example, discussing their appraisal with the supervisor [46, 52]. In designing a feedback encounter, starting with a self-assessment as feed-up, discussing with the supervisor, and identifying areas for improvement is recommended, as part of the feedback dialogue [68].

***Peer feedback*** as an organized activity allows students to develop strategies to observe and give feedback to other peers [61]. Students can act as the feedback provider or receiver, fostering understanding of critical comments and promoting evaluative judgment for their clinical practice [61]. Within clerkships, enabling the sharing of feedback information among peers allows for a better understanding and acceptance of feedback [52]. However, students can find it challenging to take on the peer assessor/feedback provider role, as they prefer to avoid social conflicts [28, 61]. Moreover, it has been described that they do not trust the judgment of their peers because they are not experts, although they know the procedures, tasks, and steps well and empathize with their peer status in the learning process [61].

***Bedside-teaching encounters*** (BTEs) provide timely feedback and are an opportunity for verbal feedback during performance [74]. Rizan et al. (2014) explored timely feedback delivered within BTEs and determined that it promotes interaction that constructively enhances learner development through various corrective strategies (e.g., question and answers, pauses, etc.). However, if the feedback given during the BTEs was general, unspecific, or open-ended, it could go unnoticed [74]. Torre et al. (2005) investigated which integrated feedback activities and clinical tasks occurred on clerkship rotations and assessed students' perceived quality in each teaching encounter [81]. The feedback activities reported were feedback on written clinical history, physical examination, differential diagnosis, oral case presentation, a daily progress note, and bedside feedback. Students considered all these feedback activities high-quality learning opportunities, but they were more likely to receive feedback when teaching was at the bedside than at other teaching locations [81].

***Case presentations*** are an opportunity for feedback within WBL contexts [67, 73]. However, both students and supervisors struggled to identify them as feedback moments, and they often dismissed questions and clarifications around case presentations as feedback [73]. Joshi (2017) identified case presentations as a way for students to ask for informal or spontaneous supervisor feedback [63].

### Organization of follow-up feedback and action plans (i.e., feedforward dimension).

Feedback that generates use and response from students is characterized by two-way communication and embedded in a dialogue [30]. Feedback must be future-focused [29], and a feedback encounter should be followed by planning the next observation [46, 87]. Follow-up feedback could be organized as a future self-assessment, reflective practice by the student, and/or a discussion with the supervisor or coach [68]. The literature describes that a lack of student interaction with teachers makes follow-up difficult [43]. According to Haffling et al. (2011), follow-up feedback sessions improve students' satisfaction with feedback compared to students who do not have follow-up sessions. In addition, these same authors reported that a second follow-up session allows verification of improved performances or confirmation that the skill was acquired [55].

Although feedback encounter forms are a recognized way of obtaining information about performance (i.e., feedback dimension), the literature does not provide many clear examples of how they may impact the feedforward phase. For example, Joshi et al. (2016) consider a feedback form with four fields (i.e., what did you do well, advise the student on what could be done to improve performance, indicate the level of proficiency, and personal details of the tutor). In this case, the supervisor highlighted what the student could improve but not how, which is the missing phase of the co-constructed action plan [63]. Whichever WBA instrument is used in clerkships to provide feedback, it should include a "next steps" box [44], and it is recommended to organize a long-term use of the WBA instrument so that those involved get used to it and improve interaction and feedback uptake [55]. RIME-based feedback (Reporting, Interpreting, Managing, Educating) is considered an interesting example, as it is perceived as helpful to students in knowing what they need to improve in their performance [44]. Hochberg (2017) implemented formative mid-clerkship assessments to enhance face-to-face feedback conversations and co-create an improvement plan [59]. Apps for structuring and storing feedback improve the amount of verbal and written feedback. In the study of Joshi et al. (2016), a reasonable proportion of students (64%) perceived that these app tools help them improve their performance during rotations [63].

Several studies indicate that an action plan as part of the follow-up feedback is essential for performance improvement and learning [46, 55, 60]. An action plan corresponds to an agreed-upon strategy for improving, confirming, or correcting performance. Bing-You et al. (2017) determined that only 12% of the articles included in their scoping review incorporated an action plan for learners [32]. Holmboe et al. (2004) reported that only 11% of the feedback sessions following a mini-CEX included an action plan [60]. Suhoyo et al. (2017) also reported that only 55% of mini-CEX encounters contained an action plan [80]. Other authors reported that action plans are not commonly offered during feedback encounters [77]. Sokol-Hessner et al. (2010) implemented feedback card comments with a space to provide written feedback and a specific action plan. In their results, 96% contained positive comments, and only 5% contained constructive comments [77]. In summary, although the recommendation is to include a “next step” box in the feedback instruments, evidence shows these items are not often used for constructive comments or action plans.

## Sociocultural factors influencing the organization of feedback processes.

Multiple sociocultural factors influence interaction in feedback encounters, promoting or hampering the productivity of the feedback processes.

### Clinical learning culture

Context impacts feedback processes [30, 82], and there are barriers to incorporating actionable feedback in the clinical learning context. The clinical learning culture is partly determined by the clinical context, which can be unpredictable [29, 46, 68], as the available patients determine learning opportunities. Supervisors are occupied by a high workload, which results in limited time or priority for teaching [35, 46, 48, 55, 68, 83], hindering students’ feedback-seeking behavior [54], and creating a challenge for the balance between patient care and student mentoring [35].

Clinical workplace culture does not always purposefully prioritize instances for feedback processes [83, 84]. This often leads to limited direct observation [55, 68] and the provision of poorly informed feedback. It is also evident that this affects trust between clinical teachers and students [52]. Supervisors consider feedback a low priority in clinical contexts [35] due to low compensation and lack of protected time [83]. In particular, lack of time appears to be the most significant and well-known barrier to frequent observation and workplace feedback [35, 43, 48, 62, 67, 83].

The clinical environment is hierarchical [68, 80] and can make students not consider themselves part of the team and feel like a burden to their supervisor [68]. This hierarchical learning environment can lead to unidirectional feedback, limit dialogue during feedback processes, and hinder the seeking, uptake, and use of feedback [67, 68]. In a learning culture where feedback is not supported, learners are less likely to want to seek it and feel motivated and engaged in their learning [83]. Furthermore, it has been identified that clinical supervisors lack the motivation to teach [48] and the intention to observe or reobserve performance [86].

In summary, the clinical context and WBL culture do not fully use the potential of a feedback process aimed at closing learning gaps. However, concrete actions shown in the literature can be taken to improve the effectiveness of feedback by organizing the learning context. For example, McGinness et al. (2022) identified that students felt more receptive to feedback when working in a safe, nonjudgmental environment [67]. Moreover, supervisors and trainees identified the learning culture as key to establishing an open feedback dialogue [73]. Students who perceive culture as supportive and formative can feel more comfortable performing tasks and more willing to receive feedback [73].

### Relationships

There is a consensus in the literature that trusting and long-term relationships improve the chances of actionable feedback. However, relationships between supervisors and students in the clinical workplace are often brief and not organized as more longitudinally [68, 83], leaving little time to establish a trustful relationship [68]. Supervisors change continuously, resulting in short interactions that limit the creation of lasting relationships over time [50, 68, 83]. In some contexts, it is common for a student to have several supervisors who have their own standards in the observation of performance [46, 56, 68, 83]. A lack of stable relationships results in students having little engagement in feedback [68]. Furthermore, in case of summative assessment programmes, the dual role of supervisors (i.e., assessing and giving feedback) makes feedback interactions perceived as summative and can complicate the relationship [83].

Repeatedly, the articles considered in this review describe that long-term and stable relationships enable the development of trust and respect [35, 62] and foster feedback-seeking behavior [35, 67] and feedback-giver behavior [39]. Moreover, constructive and positive relationships enhance students´ use of and response to feedback [30]. For example, Longitudinal Integrated Clerkships (LICs) promote stable relationships, thus enhancing the impact of feedback [83]. In a long-term trusting relationship, feedback can be straightforward and credible [87], there are more opportunities for student observation, and the likelihood of follow-up and actionable feedback improves [83]. Johnson et al. (2020) pointed out that within a clinical teacher-student relationship, the focus must be on establishing psychological safety; thus, the feedback conversations might be transformed [62].

Stable relationships enhance feedback dialogues, which offer an opportunity to co-construct learning and propose and negotiate aspects of the design of learning strategies [62].

### Students as active agents in the feedback processes

The feedback response learners generate depends on the type of feedback information they receive, how credible the source of feedback information is, the relationship between the receiver and the giver, and the relevance of the information delivered [49]. Garino (2020) noted that students who are most successful in using feedback are those who do not take criticism personally, who understand what they need to improve and know they can do so, who value and feel meaning in criticism, are not surprised to receive it, and who are motivated to seek new feedback and use effective learning strategies [52]. Successful users of feedback ask others for help, are intentional about their learning, know what resources to use and when to use them, listen to and understand a message, value advice, and use effective learning strategies. They regulate their emotions, find meaning in the message, and are willing to change [52].

Student self-efficacy influences the understanding and use of feedback in the clinical workplace. McGinness et al. (2022) described various positive examples of self-efficacy regarding feedback processes: planning feedback meetings with teachers, fostering good relationships with the clinical team, demonstrating interest in assigned tasks, persisting in seeking feedback despite the patient workload, and taking advantage of opportunities for feedback, e.g., case presentations [67].

When students are encouraged to seek feedback aligned with their own learning objectives, they promote feedback information specific to what they want to learn and improve and enhance the use of feedback [53]. McGinness et al. (2022) identified that the perceived relevance of feedback information influenced the use of feedback because students were more likely to ask for feedback if they perceived that the information was useful to them. For example, if students feel part of the clinical team and participate in patient care, they are more likely to seek feedback [17].

Learning-oriented students aim to seek feedback to achieve clinical competence at the expected level [75]; they focus on improving their knowledge and skills and on professional development [17]. Performance-oriented students aim not to fail and to avoid negative feedback [17, 75].

For effective feedback processes, including feed-up, feedback, and feedforward, the student must be feedback-oriented, i.e., active, seeking, listening to, interpreting, and acting on feedback [68]. The literature shows that feedback-oriented students are coproducers of learning [68] and are more involved in the feedback process [51]. Additionally, students who are metacognitively aware of their learning process are more likely to use feedback to reduce gaps in learning and performance [52]. For this, students must recognize feedback when it occurs and understand it when they receive it. Thus, it is important to organize training and promote feedback literacy so that students understand what feedback is, act on it, and improve the quality of feedback and their learning plans [68].

Table 5 summarizes those feedback tasks, activities, and key features of organizational aspects that enable each phase of the feedback loop based on the literature review.

**Table 5:** Summary of design aspects that facilitate the organisation of feedback and enable each feedback loop phase

	**Designing features of feedback processes to enable each feedback loop phase**
Feedup	1. Use **direct observation** for clarification of learning goals [50] 2. Encourage **dialogic feedback** for the co-construction of goals [50] 3. Focus feedback on **students’ learning needs and known performance standards** [30]
Feedback	4. Give students opportunities for **clinical practice** [35] 5. Enhance credible feedback through **direct observation** [33, 40, 46, 49, 84, 86] 6. Include **formative assessments** during authentic professional activities [46, 55] 7. Design **WBAs** during authentic tasks [39, 46, 56, 87]. The mini-CEX can provide feedback that improves students' clinical skills [58, 60] 8. Organise **self-assessments** before feedback encounters [46, 52] 9. Enhance **bedside-teaching encounters** to provide in-time feedback [74] 10. Use **questions and interpretation checks** to provide feedback on students´ performance [54], to clarify concepts and facilitate self-assessment [74] 11. Organise **oral case presentations** to improve communication skills [78] 12. Promote **benchmarking** of the same student over time (i.e., internal benchmarking), a peer, or formal guidance (i.e., external benchmarking) (e.g., a text or a guide of recommendations) [52]
Feedforward	13. Embed feedback in a two-way **conversation** [30] 14. Consider a follow-up on **direct observation** [46, 50] 15. Organise long-term use of **WBA** instruments [55] 16. Design low-stake **WBA** [31] 17. Enhance **self-assessments** when organising follow-up [68] 18. Organise formative **mini-CEX** with follow-up [39] 19. Use the **mini-CEX** as the structure for discussing the student's strengths and weaknesses and designing a written action plan [39, 80] 20. Include a **“next step” box** in the WBA instrument [44] 21. Implement a **formative mid-rotation assessment** to promote feedback conversations and co-create an action plan [59] 22. Consider **using Apps** to structure and store feedback to improve future performance [63] 23. Enable **peer feedback** for a better understanding and acceptance of feedback [52] 24. Promote **safe and non-judgmental** learning environments [67]

## Discussion

The present scoping review identified 61 papers that mapped the literature on feedback processes in the WBL environments of undergraduate health professions. This review explored how feedback processes are organized in these learning contexts using the feedback loop framework. Given the specific characteristics of feedback processes in undergraduate clinical learning, three main findings were identified on how feedback processes are being conducted in the clinical environment and how these processes could be organized to support feedback processes.

First, the literature lacks a balance between the three dimensions of the feedback loop. In this regard, most of the articles in this review focused on reporting experiences or strategies for delivering feedback information (i.e., feedback dimension). Credible and objective feedback information is based on direct observation [46] and occurs within an interaction or a dialogue [62, 88]. However, only having credible and objective information does not ensure that it will be considered, understood, used, and put into practice by the student [89].

Feedback-supporting actions aligned with goals and priorities facilitate effective feedback processes [89] because goal-oriented feedback focuses on students' learning needs [7]. In contrast, this review showed that only a minority of the studies highlighted the importance of aligning learning objectives and feedback (i.e., the feed-up dimension). To overcome this, supervisors and students must establish goals and agreements before starting clinical practice, as it allows students to measure themselves on a defined basis [90, 91] and enhances students' feedback-seeking behavior [39, 92] and responsiveness to feedback [83]. In addition, learning goals should be shared, and co-constructed, through a dialogue [50, 88, 90, 92]. In fact, relationship-based feedback models emphasize setting shared goals and plans as part of the feedback process [68].

Many of the studies acknowledge the importance of establishing an action plan and promoting the use of feedback (i.e., feedforward). However, there is yet limited insight on how to best implement strategies that support the use of action plans, improve performance and close learning gaps. In this regard, it is described that delivering feedback without perceiving changes, results in no effect or impact on learning [88]. To determine if a feedback loop is closed, observing a change in the student's response is necessary. In other words, feedback does not work without repeating the same task [68], so teachers need to observe subsequent tasks to notice changes [88]. While feedforward is fundamental to long-term performance, it is shown that more research is needed to determine effective actions to be implemented in the WBL environment to close feedback loops.

Second, there is a need for more knowledge about designing feedback activities in the WBL environment that will generate constructive feedback for learning. WBA is the most frequently reported feedback activity in clinical workplace contexts [39, 46, 56, 87]. Despite the efforts of some authors to use WBAs as a formative assessment and feedback opportunity, in several studies, a summative component of the WBA was presented as a barrier to actionable feedback [33, 56]. Students suggest separating grading from observation and using, for example, the mini-CEX in informal situations [33]. Several authors also recommend disconnecting the summative components of WBAs to avoid generating emotions that can limit the uptake and use of feedback [28, 93]. Other literature recommends purposefully designing a system of assessment using low-stakes data points for feedback and learning. Accordingly, programmatic assessment is a framework that combines both the learning and the decision-making function of assessment [94, 95]. Programmatic assessment is a practical approach for implementing low-stakes as a continuum, giving opportunities to close the gap between current and desired performance and having the student as an active agent [96]. This approach enables the incorporation of low-stakes data points that target student learning [93] and provide performance-relevant information (i.e., meaningful feedback) based on direct observations during authentic professional activities [46]. Using low-stakes data points, learners make sense of information about their performance and use it to enhance the quality of their work or performance [96–98]. Implementing multiple instances of feedback is more effective than providing it once because it promotes closing feedback loops by giving the student opportunities to understand the feedback, make changes, and see if those changes were effective [89].

Third, the support provided by the teacher is fundamental and should be built into a reliable and long-term relationship, where the teacher must take the role of coach rather than assessor, and students should develop feedback agency and be active in seeking and using feedback to improve performance. Although it is recognized that institutional efforts over the past decades have focused on training teachers to deliver feedback, clinical supervisors' lack of teaching skills is still identified as a barrier to workplace feedback [99]. In particular, research indicates that clinical teachers lack the skills to transform the information obtained from an observation into constructive feedback [100]. Students are more likely to use feedback if they consider it credible and constructive [93] and based on stable relationships [93, 99, 101]. In trusting relationships, feedback can be straightforward and credible, and the likelihood of follow-up and actionable feedback improves [83, 88]. Coaching strategies can be enhanced by teachers building an educational alliance that allows for trustworthy relationships or having supervisors with an exclusive coaching role [14, 93, 102].

Last, from a sociocultural perspective, individuals are the main actors in the learning process. Therefore, feedback impacts learning only if students engage and interact with it [11]. Thus, feedback design and student agency appear to be the main features of effective feedback processes. Accordingly, the present review identified that feedback design is a key feature for effective learning in complex environments such as WBL. Feedback in the workplace must ideally be organized and implemented to align learning outcomes, learning activities, and assessments, allowing learners to learn, practice, and close feedback loops [88]. To guide students toward performances that reflect long-term learning, an intensive formative learning phase is needed, in which multiple feedback processes are included that shape students´ further learning [103]. This design would promote student uptake of feedback for subsequent performance [1].

### Strengths and limitations

The strengths of this study are (1) the use of an established framework, the Arksey and O'Malley's framework [22]. We included the step of socializing the results with stakeholders, which allowed the team to better understand the results from another perspective and offer a realistic look. (2) Using the feedback loop as a theoretical framework strengthened the results and gave a more thorough explanation of the literature regarding feedback processes in the WBL context. (3) our team was diverse and included researchers from different disciplines as well as a librarian.

The present scoping review has several limitations. Although we adhered to the recommended protocols and methodologies, some relevant papers may have been omitted. The research team decided to select original studies and reviews of the literature for the present scoping review. This caused some articles, such as guidelines, perspectives, and narrative papers, to be excluded from the current study.

One of the inclusion criteria was a focus on undergraduate students. However, some papers that incorporated undergraduate and postgraduate participants were included, as these supported the results of this review. Most articles involved medical students. Although the authors did not limit the search to medicine, maybe some articles involving students from other health disciplines needed to be included, considering the search in other databases or journals.

## Conclusions

The results give insight in how feedback could be organized within the clinical workplace to promote feedback processes. On a small scale, i.e., in the feedback encounter between a supervisor and a learner, feedback should be organized to allow for follow-up feedback, thus working on required learning and performance goals. On a larger level, i.e., in the clerkship programme or a placement rotation, feedback should be organized through appropriate planning of subsequent tasks and activities.

More insight is needed in designing a closed loop feedback process, in which specific attention is needed in effective feedforward practices. The feedback that stimulates further action and learning requires a safe and trustful work and learning environment. Understanding the relationship between an individual and his or her environment is a challenge for determining the impact of feedback and must be further investigated within clinical WBL environments. Aligning the dimensions of feed-up, feedback and feedforward includes careful attention to teachers’ and students’ feedback literacy to assure that students can act on feedback in a constructive way. In this line, how to develop students' feedback agency within these learning environments needs further research.

### Supplementary Information

**Supplementary Material 1**.

**Supplementary Material 2.**

## References

[1] Boud D, Molloy E (2013). Rethinking models of feedback for learning: The challenge of design. Assess Eval High Educ.

[2] Henderson M, Ajjawi R, Boud D, Molloy E (2019). Identifying feedback that has impact. The Impact of Feedback in Higher Education.

[3] Winstone N, Carless D (2020). Designing effective feedback processes in higher education: A learning-focused approach.

[4] Ajjawi R, Boud D (2015). Assessment & Evaluation in Higher Education Researching feedback dialogue: an interactional analysis approach.

[5] Carless D (2019). Feedback loops and the longer-term: towards feedback spirals. Assess Eval High Educ.

[6] Sadler DR (1989). Formative assessment and the design of instructional systems. Instr Sci.

[7] Hattie J, Timperley H (2007). The Power of Feedback The Meaning of Feedback. Rev Educ Res.

[8] Zarrinabadi N, Rezazadeh M (2023). Why only feedback? Including feed up and feed forward improves nonlinguistic aspects of L2 writing. Language Teaching Research.

[9] Fisher D, Frey N (2009). Feed up, back, forward. Educ Leadersh.

[10] Reimann A, Sadler I, Sambell K (2019). What’s in a word? Practices associated with ‘feedforward’ in higher education. Assessment evaluation in higher education.

[11] Esterhazy R. Re-conceptualizing Feedback Through a Sociocultural Lens. In: Henderson M, Ajjawi R, Boud D, Molloy E, editors. The Impact of Feedback in Higher Education. Cham: Palgrave Macmillan; 2019. 10.1007/978-3-030-25112-3_5.

[12] Bransen D, Govaerts MJB, Sluijsmans DMA, Driessen EW (2020). Beyond the self: The role of co-regulation in medical students’ self-regulated learning. Med Educ.

[13] Ramani S, Könings KD, Ginsburg S, Van Der Vleuten CP (2019). Feedback Redefined: Principles and Practice. J Gen Intern Med.

[14] Atkinson A, Watling CJ, Brand PL (2022). Feedback and coaching. Eur J Pediatr..

[15] Suhoyo Y, Schonrock-Adema J, Emilia O, Kuks JBM, Cohen-Schotanus JA (2018). Clinical workplace learning: perceived learning value of individual and group feedback in a collectivistic culture. BMC Med Educ.

[16] Bowen L, Marshall M, Murdoch-Eaton D (2017). Medical Student Perceptions of Feedback and Feedback Behaviors Within the Context of the “Educational Alliance”. Acad Med.

[17] Bok HGJ, Teunissen PW, Spruijt A, Fokkema JPI, van Beukelen P, Jaarsma DADC (2013). Clarifying students’ feedback-seeking behaviour in clinical clerkships. Med Educ.

[18] Al-Kadri HM, Al-Kadi MT, Van Der Vleuten CPM (2013). Workplace-based assessment and students’ approaches to learning: A qualitative inquiry. Med Teach.

[19] Dennis AA, Foy MJ, Monrouxe LV, Rees CE (2018). Exploring trainer and trainee emotional talk in narratives about workplace-based feedback processes. Adv Health Sci Educ.

[20] Watling C, LaDonna KA, Lingard L, Voyer S, Hatala R (2016). ‘Sometimes the work just needs to be done’: socio-cultural influences on direct observation in medical training. Med Educ.

[21] Bing-You R, Hayes V, Varaklis K, Trowbridge R, Kemp H, McKelvy D (2017). Feedback for Learners in Medical Education: What is Known?. A Scoping Review Academic Medicine.

[22] Arksey H, O’Malley L (2005). Scoping studies: towards a methodological framework. Int J Soc Res Methodol.

[23] Tricco AC, Lillie E, Zarin W, O’Brien KK, Colquhoun H, Levac D (2018). PRISMA extension for scoping reviews (PRISMA-ScR): Checklist and explanation. Ann Intern Med.

[24] Colquhoun HL, Levac D, O’brien KK, Straus S, Tricco AC, Perrier L (2014). Scoping reviews: time for clarity in definition methods and reporting. J Clin Epidemiol..

[25] StArt - State of Art through Systematic Review. 2013.

[26] Levac D, Colquhoun H, O’Brien KK (2010). Scoping studies: Advancing the methodology. Implementation Science..

[27] Peters MDJ, Parker PMD, Soares CB, BPharm CMGHK (2015). Guidance for conducting systematic scoping reviews. Int J Evid Based Healthc.

[28] Bing-You R, Varaklis K, Hayes V, Trowbridge R, Kemp H, McKelvy D (2018). The Feedback Tango: An Integrative Review and Analysis of the Content of the Teacher-Learner Feedback Exchange. Acad Med.

[29] Ossenberg C, Henderson A, Mitchell M (2019). What attributes guide best practice for effective feedback? A scoping review. Adv Health Sci Educ.

[30] Spooner M, Duane C, Uygur J, Smyth E, Marron B, Murphy PJ (2022). Self -regulatory learning theory as a lens on how undergraduate and postgraduate learners respond to feedback: A BEME scoping review: BEME Guide No. 66. Med Teach.

[31] Long S, Rodriguez C, St-Onge C, Tellier PP, Torabi N, Young M (2022). Factors affecting perceived credibility of assessment in medical education: A scoping review. Adv Health Sci Educ.

[32] Bing-You R, Hayes V, Varaklis K, Trowbridge R, Kemp H, McKelvy D (2017). Feedback for Learners in Medical Education: What is Known?.

[33] Schopper H, Rosenbaum M, Axelson R (2016). “I wish someone watched me interview:” medical student insight into observation and feedback as a method for teaching communication skills during the clinical years. BMC Med Educ.

[34] Crommelinck M, Anseel F (2013). Understanding and encouraging feedback-seeking behaviour: a literature review. Med Educ.

[35] Adamson E, Kinga L, Foy L, McLeodb M, Traynor J, Watson W (2018). Feedback in clinical practice: Enhancing the students’ experience through action research. Nurse Educ Pract.

[36] Al-Mously N, Nabil NM, Al-Babtain SA (2014). Undergraduate medical students’ perceptions on the quality of feedback received during clinical rotations. Med Teach..

[37] Bates J, Konkin J, Suddards C, Dobson S, Pratt D (2013). Student perceptions of assessment and feedback in longitudinal integrated clerkships. Med Educ.

[38] Bennett AJ, Goldenhar LM, Stanford K (2006). Utilization of a Formative Evaluation Card in a Psychiatry Clerkship. Acad Psychiatry.

[39] Bok HG, Jaarsma DA, Spruijt A, Van Beukelen P, Van Der Vleuten CP, Teunissen PW (2016). Feedback-giving behaviour in performance evaluations during clinical clerkships. Med Teach..

[40] Bok HG, Teunissen PW, Spruijt A, Fokkema JP, van Beukelen P, Jaarsma DA (2013). Clarifying students’ feedback-seeking behaviour in clinical clerkships. Med Educ..

[41] Calleja P, Harvey T, Fox A, Carmichael M (2016). Feedback and clinical practice improvement: A tool to assist workplace supervisors and students. Nurse Educ Pract..

[42] Carey EG, Wu C, Hur ES, Hasday SJ, Rosculet NP, Kemp MT (2017). Evaluation of Feedback Systems for the Third-Year Surgical Clerkship. J Surg Educ.

[43] Daelmans HE, Overmeer RM, Van der Hem-Stokroos HH, Scherpbier AJ, Stehouwer CD, van der Vleuten CP (2006). In-training assessment: qualitative study of effects on supervision and feedback in an undergraduate clinical rotation. Medical education..

[44] DeWitt D, Carline J, Paauw D, Pangaro L (2008). Pilot study of a ’RIME’-based tool for giving feedback in a multi-specialty longitudinal clerkship. Med Educ.

[45] Dolan BM, O’Brien CL, Green MM (2017). Including Entrustment Language in an Assessment Form May Improve Constructive Feedback for Student Clinical Skills. Med Sci Educ.

[46] Duijn CC, Welink LS, Mandoki M, Ten Cate OT, Kremer WD, Bok HG (2017). Am I ready for it? Students’ perceptions of meaningful feedback on entrustable professional activities. Perspectives on medical education..

[47] Elnicki DM, Zalenski D (2013). Integrating medical students’ goals, self-assessment and preceptor feedback in an ambulatory clerkship. Teach Learn Med.

[48] Embo MP, Driessen EW, Valcke M, Van der Vleuten CP (2010). Assessment and feedback to facilitate self-directed learning in clinical practice of Midwifery students. Medical teacher..

[49] Eva KW, Armson H, Holmboe E, Lockyer J, Loney E, Mann K (2012). Factors influencing responsiveness to feedback: On the interplay between fear, confidence, and reasoning processes. Adv Health Sci Educ.

[50] Farrell L, Bourgeois-Law G, Ajjawi R, Regehr G (2017). An autoethnographic exploration of the use of goal oriented feedback to enhance brief clinical teaching encounters. Adv Health Sci Educ.

[51] Fernando N, Cleland J, McKenzie H, Cassar K (2008). Identifying the factors that determine feedback given to undergraduate medical students following formative mini-CEX assessments. Med Educ.

[52] Garino A (2020). Ready, willing and able: a model to explain successful use of feedback. Adv Health Sci Educ.

[53] Garner MS, Gusberg RJ, Kim AW (2014). The positive effect of immediate feedback on medical student education during the surgical clerkship. J Surg Educ.

[54] Bing-You R, Hayes V, Palka T, Ford M, Trowbridge R (2018). The Art (and Artifice) of Seeking Feedback: Clerkship Students’ Approaches to Asking for Feedback. Acad Med.

[55] Haffling AC, Beckman A, Edgren G (2011). Structured feedback to undergraduate medical students: 3 years’ experience of an assessment tool. Medical teacher..

[56] Harrison CJ, Könings KD, Dannefer EF, Schuwirth LWTT, Wass V, van der Vleuten CPMM (2016). Factors influencing students’ receptivity to formative feedback emerging from different assessment cultures. Perspect Med Educ.

[57] Harrison CJ, Könings KD, Schuwirth LW, Wass V, Van der Vleuten CP, Konings KD (2017). Changing the culture of assessment: the dominance of the summative assessment paradigm. BMC medical education..

[58] Harvey P, Radomski N, O’Connor D (2013). Written feedback and continuity of learning in a geographically distributed medical education program. Medical teacher..

[59] Hochberg M, Berman R, Ogilvie J, Yingling S, Lee S, Pusic M (2017). Midclerkship feedback in the surgical clerkship: the “Professionalism, Reporting, Interpreting, Managing, Educating, and Procedural Skills” application utilizing learner self-assessment. Am J Surg.

[60] Holmboe ES, Yepes M, Williams F, Huot SJ (2004). Feedback and the mini clinical evaluation exercise. Journal of general internal medicine..

[61] Tai JHM, Canny BJ, Haines TP, Molloy EK (2016). The role of peer-assisted learning in building evaluative judgement: opportunities in clinical medical education. Adv Health Sci Educ.

[62] Johnson CE, Keating JL, Molloy EK (2020). Psychological safety in feedback: What does it look like and how can educators work with learners to foster it?. Med Educ.

[63] Joshi A, Generalla J, Thompson B, Haidet P (2017). Facilitating the Feedback Process on a Clinical Clerkship Using a Smartphone Application. Acad Psychiatry.

[64] Kiger ME, Riley C, Stolfi A, Morrison S, Burke A, Lockspeiser T (2020). Use of Individualized Learning Plans to Facilitate Feedback Among Medical Students. Teach Learn Med.

[65] Kogan J, Shea J (2008). Implementing feedback cards in core clerkships. Med Educ.

[66] Lefroy J, Walters B, Molyneux A, Smithson S (2021). Can learning from workplace feedback be enhanced by reflective writing? A realist evaluation in UK undergraduate medical education. Educ Prim Care.

[67] McGinness HT, Caldwell PHY, Gunasekera H, Scott KM (2022). ‘Every Human Interaction Requires a Bit of Give and Take’: Medical Students’ Approaches to Pursuing Feedback in the Clinical Setting. Teach Learn Med.

[68] Noble C, Billett S, Armit L, Collier L, Hilder J, Sly C (2020). ``It’s yours to take{’’}: generating learner feedback literacy in the workplace. Adv Health Sci Educ.

[69] Ogburn T, Espey E (2003). The R-I-M-E method for evaluation of medical students on an obstetrics and gynecology clerkship. Am J Obstet Gynecol.

[70] Po O, Reznik M, Greenberg L (2007). Improving a medical student feedback with a clinical encounter card. Ambul Pediatr.

[71] Parkes J, Abercrombie S, McCarty T, Parkes J, Abercrombie S, McCarty T (2013). Feedback sandwiches affect perceptions but not performance. Adv Health Sci Educ.

[72] Paukert JL, Richards ML, Olney C (2002). An encounter card system for increasing feedback to students. Am J Surg.

[73] Rassos J, Melvin LJ, Panisko D, Kulasegaram K, Kuper A (2019). Unearthing Faculty and Trainee Perspectives of Feedback in Internal Medicine: the Oral Case Presentation as a Model. J Gen Intern Med.

[74] Rizan C, Elsey C, Lemon T, Grant A, Monrouxe L (2014). Feedback in action within bedside teaching encounters: a video ethnographic study. Med Educ.

[75] Robertson AC, Fowler LC (2017). Medical student perceptions of learner-initiated feedback using a mobile web application. Journal of medical education and curricular development..

[76] Scheidt PC, Lazoritz S, Ebbeling WL, Figelman AR, Moessner HF, Singer JE (1986). Evaluation of system providing feedback to students on videotaped patient encounters. J Med Educ..

[77] Sokol-Hessner L, Shea J, Kogan J (2010). The open-ended comment space for action plans on core clerkship students’ encounter cards: what gets written?. Acad Med.

[78] Sox CM, Dell M, Phillipi CA, Cabral HJ, Vargas G, Lewin LO (2014). Feedback on oral presentations during pediatric clerkships: a randomized controlled trial. Pediatrics.

[79] Spickard A, Gigante J, Stein G, Denny JC (2008). Automatic capture of student notes to augment mentor feedback and student performance on patient write-ups. J Gen Intern Med.

[80] Suhoyo Y, Van Hell EA, Kerdijk W, Emilia O, Schönrock-Adema J, Kuks JB (2017). nfluence of feedback characteristics on perceived learning value of feedback in clerkships: does culture matter?. BMC medical education.

[81] Torre DM, Simpson D, Sebastian JL, Elnicki DM (2005). Learning/feedback activities and high-quality teaching: perceptions of third-year medical students during an inpatient rotation. Acad Med.

[82] Urquhart LM, Ker JS, Rees CE (2018). Exploring the influence of context on feedback at medical school: a video-ethnography study. Adv Health Sci Educ.

[83] Watling C, Driessen E, van der Vleuten C, Lingard L (2014). Learning culture and feedback: an international study of medical athletes and musicians. Med Educ.

[84] Watling C, Driessen E, van der Vleuten C, Vanstone M, Lingard L (2013). Beyond individualism: Professional culture and its influence on feedback. Med Educ.

[85] Soemantri D, Dodds A, Mccoll G (2018). Examining the nature of feedback within the Mini Clinical Evaluation Exercise (Mini-CEX): an analysis of 1427 Mini-CEX assessment forms. GMS J Med Educ.

[86] Van De Ridder JMMM, Stokking KM, McGaghie WC, ten Cate OTJ, van der Ridder JM, Stokking KM (2008). What is feedback in clinical education?. Med Educ.

[87] van de Ridder JMMM, McGaghie WC, Stokking KM, ten Cate OTJJ (2015). Variables that affect the process and outcome of feedback, relevant for medical training: a meta-review. Med Educ.

[88] Boud D (2015). Feedback: ensuring that it leads to enhanced learning. Clin Teach.

[89] Brehaut J, Colquhoun H, Eva K, Carrol K, Sales A, Michie S (2016). Practice feedback interventions: 15 suggestions for optimizing effectiveness. Ann Intern Med.

[90] Ende J (1983). Feedback in clinical medical education. J Am Med Assoc.

[91] Cantillon P, Sargeant J (2008). Giving feedback in clinical settings. Br Med J..

[92] Norcini J, Burch V (2007). Workplace-based assessment as an educational tool: AMEE Guide No. 31. Med Teach..

[93] Watling CJ, Ginsburg S (2019). Assessment, feedback and the alchemy of learning. Med Educ.

[94] van der Vleuten CPM, Schuwirth LWT, Driessen EW, Dijkstra J, Tigelaar D, Baartman LKJ (2012). A model for programmatic assessment fit for purpose. Med Teach.

[95] Schuwirth LWT, der Vleuten CPM (2011). Programmatic assessment: from assessment of learning to assessment for learning. Med Teach.

[96] Schut S, Driessen E, van Tartwijk J, van der Vleuten C, Heeneman S (2018). Stakes in the eye of the beholder: an international study of learners’ perceptions within programmatic assessment. Med Educ.

[97] Henderson M, Boud D, Molloy E, Dawson P, Phillips M, Ryan T, Mahoney MP. Feedback for learning. Closing the assessment loop. Framework for effective learning. Canberra, Australia: Australian Government, Department for Education and Training; 2018.

[98] Heeneman S, Pool AO, Schuwirth LWT, van der Vleuten CPM, Driessen EW, Oudkerk A (2015). The impact of programmatic assessment on student learning: theory versus practice. Med Educ.

[99] Lefroy J, Watling C, Teunissen P, Brand P, Watling C (2015). Guidelines: the do’s, don’ts and don’t knows of feedback for clinical education. Perspect Med Educ.

[100] Ramani S, Krackov SK (2012). Twelve tips for giving feedback effectively in the clinical environment. Med Teach.

[101] Telio S, Ajjawi R, Regehr G (2015). The, “Educational Alliance” as a Framework for Reconceptualizing Feedback in Medical Education. Acad Med.

[102] Lockyer J, Armson H, Könings KD, Lee-Krueger RC, des Ordons AR, Ramani S (2020). In-the-Moment Feedback and Coaching: Improving R2C2 for a New Context. J Grad Med Educ.

[103] Black P, Wiliam D (2009). Developing the theory of formative assessment. Educ Assess Eval Account.

