# Supplementary material for: Designing feedback processes in the workplace-based learning of undergraduate health professions education: a scoping review
Source: BMC Med Educ. 2024 Apr 23;24:440. doi: 10.1186/s12909-024-05439-6 (PMC11036781; doi:10.1186/s12909-024-05439-6)
Supplement: Supplementary file 1 — Supplementary Material 1. [file 12909_2024_5439_MOESM1_ESM.docx]

**Appendix 1**

**Glossary of terms**

**Feed-up:** Refers to the component that provides clarity and guidance regarding the goals, expectations, and criteria for success to learners.

**Feedback*:*** *“information about the gap between the actual level and the reference level of a system parameter which is used to alter the gap in some way”* (Ramaprasad 1983). “*Feedback* *is where the learner makes sense of performance-relevant information to promote their learning”* (Henderson et al. 2019).

**Formative feedback:** “i*nformation communicated to the learner that is intended to modify the learner’s thinking or behaviour for the purpose of improving learning”* (*Feedback - MeSH - NCBI*).

**Feedback processes:** “*involve information which usually comes from a peer, a teacher, or oneself. They also involve sense making when students engage with and interpret comments they have received”* (Carless 2016).

**Feedback loop:** Process composed by three fundamental stages: the feedup, the feedback, and the feedforward.

**Feedforward:** *“Specifically associated with ‘nested tasks’, timed and designed to elicit input, judgements of students’ own performance and that of others, and improved performance on subsequent tasks”* (Reimann et al. 2019).

**Workplace-based learning:** *“Interaction between a qualified health care professional or patient and a student healthcare professional that takes place in the clinical workplace rather than in a classroom”* (Bird et al. 2015).

**Undergraduate medical education**: Is the period of medical education in a medical school. Usually includes the clerkships which is the clinical practice phase.

**Clinical clerkship:** Undergraduate education programs for second-, third- , and fourth-year students in health sciences in which the students receive clinical training and experience in teaching hospitals or affiliated health centers.

**Health professions:** Professions or other business activities directed to the cure and prevention of disease.

**References**

Bird KS, Newman M, Hargreaves K, Sawtell M. 2015. Workplace-based learning for undergraduate and pre-registration healthcare professionals: a systematic map of the research literature.

Carless D. 2016. Feedback as Dialogue. Encycl Educ Philos Theory. doi:10.1007/978-981-287-532-7_389-1.

feedback - MeSH - NCBI.

Henderson M, Ajjawi R, Boud D, Molloy E. 2019. The Impact of Feedback in Higher Education. Springer International Publishing.

Ramaprasad A. 1983. On the definition of feedback. Behav Sci. 28(1):4–13. doi:10.1002/bs.3830280103.

Reimann A, Sadler I, Sambell K. 2019. What’s in a word? Practices associated with ‘feedforward’ in higher education . Assess Eval High Educ. 44(8):1279–1290. doi:10.1080/02602938.2019.1600655.
