# Supplementary material for: Designing feedback processes in the workplace-based learning of undergraduate health professions education: a scoping review
Source: BMC Med Educ. 2024 Apr 23;24:440. doi: 10.1186/s12909-024-05439-6 (PMC11036781; doi:10.1186/s12909-024-05439-6)
Supplement: Supplementary file 2 — Supplementary Material 2. [file 12909_2024_5439_MOESM2_ESM.docx]

| PubMed Medline | | |
| --- | --- | --- |
| 1  Feedback | ("Formative Feedback"[Mesh] OR feedback) | 202,011 |
| 2  Workplace, clinical clerkship | ("Workplace"[Mesh] OR workplace OR "Clinical Clerkship"[Mesh] OR clerkship) | 77,937 |
| 3  Undergraduate students | (("Education, Medical, Undergraduate"[Mesh] OR undergraduate health profession*) OR (learner* medical education)) | 41,018 |
| ("Formative Feedback"[Mesh] OR feedback) AND ("Workplace"[Mesh] OR workplace OR "Clinical Clerkship"[Mesh] OR clerkship) AND (("Education, Medical, Undergraduate"[Mesh] OR undergraduate health profession*) OR (learner* medical education)) | | 529 |

**Appendix 2**

**Search strategy**

**November 2022**
